# Association between preoperative nutritional status and anastomotic leakage in patients undergoing rectal cancer surgery

**DOI:** 10.3389/fnut.2026.1932994

**Published:** 2026-10-28

**Authors:** Yan-Jun Lu, Ling-Hong Guo, Peng-Cheng Wang

**Affiliations:** Department of Colorectal Surgery, Shanxi Province Cancer Hospital; Shanxi Hospital Affiliated to Cancer Hospital, Chinese Academy of Medical Sciences; Cancer Hospital Affiliated to Shanxi Medical University, Taiyuan, Shanxi, China

**Keywords:** anastomotic leakage, C-reactive protein, NRS-2002, nutritional status, prognostic nutritional index, propensity score matching, rectal cancer

## Abstract

**Background:**

Anastomotic leakage is a clinically important complication after rectal cancer surgery, and preoperative nutritional and inflammatory status may be associated with anastomotic healing. This study aimed to evaluate the association between preoperative nutritional status, particularly the prognostic nutritional index (PNI), and postoperative anastomotic leakage in patients undergoing rectal cancer surgery.

**Methods:**

This retrospective study included consecutive eligible patients who underwent rectal cancer surgery between January 2023 and December 2025. Anastomotic leakage within 30 days was identified according to International Study Group of Rectal Cancer criteria. Preoperative nutritional status was primarily assessed using PNI, with Nutritional Risk Screening 2002 (NRS-2002), serum nutritional markers, and C-reactive protein (CRP) evaluated as complementary measures. Propensity score matching (PSM), multivariable logistic regression, unmatched-cohort analysis, sequential adjustment, subgroup analyses, and receiver operating characteristic analysis were performed.

**Results:**

Among 2,468 patients, 308 developed anastomotic leakage. After 1:1 PSM, 308 patients with leakage and 308 without leakage were analyzed. Mean PNI was lower in patients with leakage (43.2 ± 5.1 vs. 46.8 ± 4.9; *p* < 0.001). Each 1-point increase in PNI was associated with lower adjusted odds of leakage (odds ratio [OR], 0.898; 95% confidence interval [CI], 0.861–0.936; *p* < 0.001), with a similar estimate in the unmatched cohort (OR, 0.904; 95% CI, 0.882–0.927). NRS-2002 ≥ 3 was associated with higher odds of leakage (OR, 1.72; 95% CI, 1.12–2.65). Additional adjustment for CRP attenuated but did not eliminate the PNI association (OR, 0.907; 95% CI, 0.870–0.945). The optimal PNI cutoff was 45.5, with an area under the receiver operating characteristic curve of 0.646, sensitivity of 62.7%, and specificity of 60.8%.

**Conclusion:**

Lower preoperative PNI was independently associated with higher odds of postoperative anastomotic leakage after rectal cancer surgery. PNI may provide a readily available adjunct to multidimensional preoperative risk assessment but should not be considered a stand-alone predictor.

## 1 Introduction

Colorectal cancer remains a major global health burden and continues to rank among the leading causes of cancer incidence and mortality worldwide (1). Rectal cancer accounts for a substantial proportion of colorectal malignancies and frequently requires technically demanding pelvic surgery as a central component of curative treatment (2, 3). Despite ongoing advances in total mesorectal excision, minimally invasive techniques, perioperative care pathways, and neoadjuvant treatment strategies, postoperative morbidity remains clinically relevant, particularly in patients undergoing sphincter-preserving resection with colorectal or coloanal anastomosis (4, 5). Among postoperative complications, anastomotic leakage is one of the most consequential events after rectal cancer surgery. It is associated with reintervention, prolonged hospitalization, delayed recovery, and impaired subsequent treatment delivery (6, 7). Recent studies have also highlighted the broader implications of anastomotic leakage for long-term functional and oncologic outcomes, reinforcing the importance of accurate perioperative risk stratification and timely preventive strategies (8, 9). At the same time, contemporary predictive modeling studies indicate that anastomotic leakage remains difficult to anticipate using technical variables alone, suggesting that host-related factors deserve closer attention (10–12).

Preoperative nutritional status has emerged as a potentially important determinant of short-term outcomes in colorectal surgery. Patients with rectal cancer are particularly vulnerable to nutritional impairment because of tumor-related anorexia, altered bowel habits, catabolic inflammation, bowel obstruction, treatment-related toxicity, and reduced oral intake during neoadjuvant therapy (13, 14). Recent perioperative colorectal guidelines recommend prehabilitation and preoperative nutritional support for selected patients, reflecting growing recognition that nutritional optimization may improve surgical resilience (15, 16). In parallel, recent reviews of nutritional prehabilitation and multimodal prehabilitation have emphasized that nutritional screening and targeted intervention should be incorporated into perioperative management rather than treated as secondary considerations (17, 18).

Within this context, the PNI, derived from serum albumin concentration and peripheral lymphocyte count, has gained increasing attention as a readily available composite marker reflecting nutritional, immunologic, and inflammatory status (19). Recent studies in colorectal and rectal cancer have associated lower PNI with postoperative complications, including anastomotic leakage, as well as less favorable oncologic outcomes (20). Other nutritional indicators and validated screening tools may provide complementary information, while the inflammation-sensitive components of PNI raise the possibility that systemic inflammatory status contributes to the observed associations (21, 22). However, available evidence remains heterogeneous, and uncertainty persists regarding whether the association between PNI and anastomotic leakage remains consistent after rigorous control of baseline and inflammatory confounding and whether PNI alone provides clinically useful discrimination. Therefore, the present study aimed to evaluate the association between preoperative nutritional status and postoperative anastomotic leakage after rectal cancer surgery, with PNI as the primary exposure, while additionally examining complementary nutritional and inflammatory measures, the robustness of the association across analytical approaches, and the discriminatory performance of PNI.

## 2 Methods

### 2.1 Study design

This retrospective study included consecutive eligible patients who underwent surgery for rectal cancer at our institution between January 2023 and December 2025. The source cohort comprised all eligible patients during the study period. Patients who developed postoperative anastomotic leakage were classified into the leakage group, whereas those without anastomotic leakage constituted the source non-leakage group. To construct a comparison cohort with improved covariate balance, patients without leakage were subsequently matched 1:1 to patients with leakage using propensity score matching (PSM). The propensity score-matched cohort was used for the primary comparative and multivariable analyses, whereas the original unmatched cohort was retained for complementary analyses, including PNI quartile analyses and analyses evaluating the robustness and discriminatory performance of PNI. Eligible patients were adults with histologically confirmed rectal adenocarcinoma who underwent surgical resection at our institution during the study period and had the required preoperative nutritional, laboratory, perioperative, and outcome data available. Patients were excluded if they underwent emergency surgery; underwent Hartmann procedure or another procedure without primary anastomosis; had recurrent rectal cancer or synchronous malignancies; had active preoperative infection or severe inflammatory disease; had hematological disorders or severe hepatic or renal dysfunction that could substantially affect nutritional or laboratory assessment; or had incomplete medical records. The study protocol was reviewed and approved by the Ethics Committee of our hospital. All procedures were conducted in accordance with relevant guidelines and regulations and the Declaration of Helsinki. All data were de-identified and handled confidentially to protect participant privacy.

### 2.2 Definition and outcome assessment of anastomotic leakage

The primary outcome was postoperative anastomotic leakage occurring within 30 days after surgery. Anastomotic leakage was defined according to the criteria of the International Study Group of Rectal Cancer (ISREC) as a defect in the integrity of the intestinal wall at the anastomotic site resulting in communication between the intra- and extraluminal compartments. Anastomotic leakage was identified retrospectively from the medical records according to the ISREC criteria and was considered present when supported by one or more clinical, laboratory, radiological, endoscopic, or operative findings. Clinical findings included fever, abdominal pain, signs of peritoneal irritation, or purulent or feculent drainage. Laboratory findings included abnormalities suggestive of postoperative infection or inflammation. Radiological evidence on computed tomography included pelvic fluid collection, extraluminal gas, abscess formation, or contrast extravasation adjacent to the anastomosis. Additional confirmation could be obtained from reoperation, image-guided drainage, or endoscopic examination. Outcome ascertainment was based on inpatient medical records and short-term postoperative follow-up records, and only leakage events documented within 30 days after surgery were included in the analysis.

### 2.3 Preoperative nutritional and inflammatory assessment

Preoperative nutritional status was primarily assessed using the prognostic nutritional index (PNI). Laboratory parameters used to calculate PNI were obtained from routine preoperative blood tests, using the most recent measurements available within 24–72 h before surgery. PNI was calculated as follows: PNI = serum albumin (g/L) + 5 × peripheral blood lymphocyte count (×10^9^/L). In addition to PNI, serum albumin, prealbumin, total protein, body mass index (BMI), peripheral blood lymphocyte count, and preoperative anemia status were evaluated as complementary indicators of preoperative nutritional status. Preoperative C-reactive protein (CRP) was additionally evaluated as an inflammatory marker because serum albumin and lymphocyte count may also vary in association with systemic inflammatory status. Nutritional Risk Screening 2002 (NRS-2002) scores documented as part of routine preoperative admission assessment were retrospectively extracted from the medical records. NRS-2002 was analyzed both as a continuous score and categorically using the conventional threshold of <3 versus ≥3. Continuous and categorical NRS-2002 measures were evaluated in separate regression models.

### 2.4 Propensity score matching

PSM was used to reduce measured baseline covariate imbalance between patients with and without postoperative anastomotic leakage. Propensity scores were estimated using a logistic regression model including demographic, clinical, tumor-related, and treatment-related covariates selected *a priori* on the basis of clinical relevance and data availability. These variables included age, sex, BMI, tumor location, neoadjuvant chemoradiotherapy, surgical approach, American Society of Anesthesiologists (ASA) physical status classification, diabetes mellitus, protective stoma creation, and tumor stage. PNI, its individual components, other nutritional indicators, CRP, and NRS-2002 were not included in the propensity score model because PNI was the primary nutritional exposure of interest and the related nutritional and inflammatory measures were evaluated separately in subsequent analyses. Patients with anastomotic leakage were matched 1:1 to patients without leakage using nearest-neighbor matching without replacement, with a caliper width of 0.2 standard deviations of the logit of the propensity score. Covariate balance before and after matching was assessed using standardized mean differences (SMDs), with an absolute SMD < 0.10 considered indicative of acceptable balance. Because matching selected a subset of patients from the source non-leakage cohort, the distributions of the matching covariates were also compared between the complete source non-leakage cohort and the matched non-leakage controls using SMDs to characterize differences introduced by the matching process.

### 2.5 Collection of clinical and perioperative data

Clinical and perioperative data were retrospectively retrieved from the institutional electronic medical record system, operative reports, anesthetic records, laboratory database, pathological reports, nutritional screening records, and postoperative follow-up records. Extracted data included demographic characteristics, comorbidities, tumor-related characteristics, treatment- and procedure-related variables, and preoperative laboratory measurements. Demographic variables included age, sex, height, body weight, BMI, smoking history, and alcohol consumption history. Comorbidities and baseline clinical conditions included hypertension, diabetes mellitus, coronary artery disease, chronic obstructive pulmonary disease, chronic liver disease, chronic kidney disease, ASA physical status classification, and preoperative anemia status. Tumor-related variables included the distance from the lower tumor margin to the anal verge, maximum tumor diameter, histological differentiation, tumor–node–metastasis (TNM) stage, T stage, N stage, presence of bowel obstruction, and preoperative carcinoembryonic antigen level. Treatment- and procedure-related variables included neoadjuvant chemoradiotherapy, surgical approach, operative time, intraoperative blood loss, perioperative blood transfusion, protective stoma creation, anastomotic technique, anastomotic level, and pelvic drainage. Anastomotic level was recorded according to the distance of the anastomosis from the anal verge, with an anastomotic level ≤5 cm categorized as low. Preoperative laboratory variables included white blood cell count, neutrophil count, lymphocyte count, hemoglobin, platelet count, serum albumin, prealbumin, total protein, CRP, serum creatinine, blood glucose, and total cholesterol. For laboratory variables, the most recent measurement obtained within 24–72 h before surgery was used.

### 2.6 Statistical analysis

Statistical analyses were performed using IBM SPSS Statistics, version 30.0 (IBM Corp., Armonk, NY, USA). Continuous variables were summarized as mean ± standard deviation or median (interquartile range), as appropriate, and categorical variables as number (percentage). Between-group comparisons were performed using the independent-samples *t* test or Mann–Whitney U test for continuous variables and the chi-square test for categorical variables. Standardized mean differences (SMDs) were used to assess covariate balance, with an absolute SMD < 0.10 indicating acceptable balance. Logistic regression was used to estimate associations with postoperative anastomotic leakage, with results reported as odds ratios (ORs) and 95% confidence intervals (CIs). The primary multivariable analysis was performed in the propensity score-matched cohort with PNI modeled as a continuous variable. In the original cohort, PNI was additionally categorized into quartiles, with the highest quartile as the reference. Serum albumin, prealbumin, and preoperative anemia were evaluated in separate models as alternative nutritional indicators. NRS-2002 was analyzed separately as a continuous variable and as <3 versus ≥3. The primary multivariable analysis was also repeated in the unmatched cohort. Sequential regression models were used to examine the association of PNI under increasing covariate adjustment, with CRP additionally included in the final model. E-values were calculated for the PNI estimates and the confidence limits closest to the null. Receiver operating characteristic analysis was performed in the original cohort, and the optimal PNI cutoff was determined by the maximum Youden index. Subgroup analyses were conducted for neoadjuvant chemoradiotherapy, tumor location, protective stoma creation, diabetes mellitus, and tumor stage, with interaction tests used to assess heterogeneity. The association between protective stoma creation and leakage was also examined according to anastomotic level (≤5 vs. >5 cm). All tests were two-sided, with *p* < 0.05 considered statistically significant.

## 3 Results

### 3.1 Patient selection and matching results

During the study period, 2,610 patients with rectal adenocarcinoma who underwent surgical resection at our institution were screened for eligibility. After application of the predefined eligibility criteria, 142 patients were excluded: 24 who underwent emergency surgery, 39 who underwent Hartmann procedure or another procedure without primary anastomosis, 18 with recurrent rectal cancer or synchronous malignancies, 16 with active preoperative infection or severe inflammatory disease, 21 with hematological disorders or severe hepatic or renal dysfunction that could substantially affect nutritional assessment, and 24 with incomplete medical records. The original cohort therefore comprised 2,468 patients, including 308 with postoperative anastomotic leakage and 2,160 without leakage. After 1:1 PSM, the matched cohort comprised 308 patients with leakage and 308 patients without leakage.

### 3.2 Baseline characteristics before and after propensity score matching

Before PSM, several baseline characteristics differed between patients with and without anastomotic leakage. The largest imbalances were observed for age, BMI, tumor location, surgical approach, ASA physical status classification, diabetes mellitus, protective stoma creation, and tumor stage, with SMDs ranging from 0.195 to 0.362 for most of these variables. After 1:1 matching, 308 patients with anastomotic leakage and 308 matched patients without leakage were included. All prespecified matching covariates achieved SMDs below 0.10, with post-matching SMDs ranging from 0.026 to 0.063, indicating substantially improved covariate balance between the two groups (Table 1).

**Table 1:** Baseline characteristics before and after propensity score matching.

Variable	Before PSM leakage (*n* = 308)	Before PSM non-leakage (*n* = 2,160)	SMD	After PSM leakage (*n* = 308)	After PSM non-leakage (*n* = 308)	SMD
Age, years	66.8 ± 9.1	63.4 ± 9.7	0.362	66.8 ± 9.1	66.5 ± 8.9	0.033
Male sex, *n* (%)	209 (67.9)	1,364 (63.1)	0.099	209 (67.9)	205 (66.6)	0.028
BMI, kg/m^2^	23.3 ± 3.2	24.2 ± 3.3	0.277	23.3 ± 3.2	23.5 ± 3.1	0.063
Tumor location, *n* (%)			0.215			0.039
Low rectum	156 (50.6)	864 (40.0)		156 (50.6)	150 (48.7)	
Mid rectum	108 (35.1)	842 (39.0)		108 (35.1)	112 (36.4)	
High rectum	44 (14.3)	454 (21.0)		44 (14.3)	46 (14.9)	
Neoadjuvant chemoradiotherapy, *n* (%)	118 (38.3)	629 (29.1)	0.195	118 (38.3)	114 (37.0)	0.027
Surgical approach, *n* (%)			0.212			0.034
Open surgery	86 (27.9)	410 (19.0)		86 (27.9)	82 (26.6)	
Laparoscopic surgery	198 (64.3)	1,512 (70.0)		198 (64.3)	203 (65.9)	
Robotic surgery	24 (7.8)	238 (11.0)		24 (7.8)	23 (7.5)	
ASA physical status classification, *n* (%)			0.216			0.033
I–II	171 (55.5)	1,426 (66.0)		171 (55.5)	166 (53.9)	
III–IV	137 (44.5)	734 (34.0)		137 (44.5)	142 (46.1)	
Diabetes mellitus, *n* (%)	85 (27.6)	410 (19.0)	0.205	85 (27.6)	81 (26.3)	0.029
Protective stoma creation, *n* (%)	142 (46.1)	756 (35.0)	0.228	142 (46.1)	138 (44.8)	0.026
Tumor stage, *n* (%)			0.257			0.026
Stage I	52 (16.9)	540 (25.0)		52 (16.9)	55 (17.9)	
Stage II	116 (37.7)	907 (42.0)		116 (37.7)	113 (36.7)	
Stage III	140 (45.5)	713 (33.0)		140 (45.5)	140 (45.5)	

ASA, American Society of Anesthesiologists; BMI, body mass index; PSM, propensity score matching; SMD, standardized mean difference.

Because matching selected a subset of the 2,160 patients without leakage, the characteristics of the 308 matched controls were also compared with those of the source non-leakage cohort. Differences remained for several characteristics, including age (SMD = 0.333), BMI (SMD = 0.219), ASA III–IV status (SMD = 0.249), protective stoma creation (SMD = 0.201), and tumor stage (SMD = 0.257), reflecting the selection of controls with characteristics more comparable to those of the leakage group (Supplementary Table S1).

### 3.3 Preoperative nutritional and inflammatory characteristics

In the matched cohort, patients with anastomotic leakage had a lower mean preoperative PNI than those without leakage (43.2 ± 5.1 vs. 46.8 ± 4.9, *p* < 0.001). Serum albumin, prealbumin, total protein, and peripheral lymphocyte count were also lower in the leakage group, whereas preoperative anemia was more frequent (44.8% vs. 29.9%, *p* < 0.001). Preoperative CRP concentrations were higher among patients with leakage [median, 6.4 mg/L (IQR, 3.3–11.8)] than among those without leakage [4.8 mg/L (IQR, 2.6–8.4); *p* < 0.001]. NRS-2002 scores were also higher in the leakage group [median, 2 (IQR, 1–3) vs. 1 (IQR, 1–2), *p* < 0.001], and an NRS-2002 score ≥3 was present in 26.6 and 15.6% of patients, respectively (*p* = 0.001). BMI did not differ significantly between the groups after matching (Table 2).

**Table 2:** Preoperative nutritional and inflammatory characteristics after propensity score matching.

Variable	Leakage group (*n* = 308)	Non-leakage group (*n* = 308)	Test statistic	*p* value
PNI	43.2 ± 5.1	46.8 ± 4.9	*t* = −8.93	<0.001
BMI, kg/m^2^	23.3 ± 3.2	23.5 ± 3.1	*t* = −0.79	0.431
Serum albumin, g/L	36.8 ± 4.2	39.1 ± 4.0	*t* = −6.96	<0.001
Prealbumin, mg/L	192.6 ± 41.7	214.8 ± 43.5	*t* = −6.47	<0.001
Total protein, g/L	63.9 ± 6.8	66.1 ± 6.5	*t* = −4.10	<0.001
Lymphocyte count, ×10^9^/L	1.32 ± 0.41	1.54 ± 0.45	*t* = −6.34	<0.001
Preoperative anemia, *n* (%)	138 (44.8)	92 (29.9)	*χ*^2^ = 14.68	<0.001
C-reactive protein, mg/L, median (IQR)	6.4 (3.3–11.8)	4.8 (2.6–8.4)	*Z* = −4.01	<0.001
NRS-2002 score, median (IQR)	2 (1–3)	1 (1–2)	*Z* = −4.35	<0.001
NRS-2002 ≥ 3, *n* (%)	82 (26.6)	48 (15.6)	*χ*^2^ = 11.27	0.001

BMI, body mass index; IQR, interquartile range; NRS-2002, Nutritional Risk Screening 2002; PNI, prognostic nutritional index.

In multivariable analyses of NRS-2002, each 1-point higher score was associated with higher odds of anastomotic leakage (adjusted OR, 1.31; 95% CI, 1.12–1.54; *p* < 0.001). When NRS-2002 was analyzed categorically, a score ≥3 was also associated with higher odds of leakage compared with a score <3 (adjusted OR, 1.72; 95% CI, 1.12–2.65; *p* = 0.013) (Supplementary Table S2).

### 3.4 Association between PNI quartiles and anastomotic leakage

In the original cohort of 2,468 patients, PNI was divided into four quartiles of 617 patients each. The proportion of patients with anastomotic leakage decreased across increasing PNI quartiles, from 19.0% in Q1 (PNI ≤ 43.1) to 13.9% in Q2, 9.9% in Q3, and 7.1% in Q4 (PNI ≥ 49.5). Using Q4 as the reference category, the adjusted odds of leakage were higher in Q1 (adjusted OR, 2.56; 95% CI, 1.75–3.75; *p* < 0.001) and Q2 (adjusted OR, 1.83; 95% CI, 1.24–2.70; *p* = 0.002). The corresponding estimate for Q3 was smaller and was not statistically significant (adjusted OR, 1.31; 95% CI, 0.87–1.97; *p* = 0.198) (Table 3).

**Table 3:** Association between preoperative PNI quartiles and anastomotic leakage in the original cohort.

PNI quartile	PNI range	Total, *n*	AL, *n* (%)	Non-AL, *n* (%)	Crude OR (95% CI)	Crude *p* value	Adjusted OR (95% CI)	Adjusted *p* value
Q1	≤43.1	617	117 (19.0)	500 (81.0)	3.05 (2.11–4.40)	<0.001	2.56 (1.75–3.75)	<0.001
Q2	43.2–46.2	617	86 (13.9)	531 (86.1)	2.11 (1.44–3.09)	<0.001	1.83 (1.24–2.70)	0.002
Q3	46.3–49.4	617	61 (9.9)	556 (90.1)	1.43 (0.95–2.14)	0.084	1.31 (0.87–1.97)	0.198
Q4	≥49.5	617	44 (7.1)	573 (92.9)	1.00 (reference)	—	1.00 (reference)	—

AL, anastomotic leakage; CI, confidence interval; OR, odds ratio; PNI, prognostic nutritional index.

### 3.5 Multivariable analysis in the propensity score-matched cohort

In the matched cohort, higher preoperative PNI remained associated with lower odds of postoperative anastomotic leakage after multivariable adjustment. Each 1-point increase in PNI corresponded to an adjusted OR of 0.898 (95% CI, 0.861–0.936; *p* < 0.001). Preoperative anemia was associated with higher odds of leakage (adjusted OR, 1.756; 95% CI, 1.214–2.540; *p* = 0.003), whereas greater tumor distance from the anal verge was associated with lower odds (adjusted OR per 1-cm increase, 0.839; 95% CI, 0.765–0.921; *p* < 0.001). Longer operative time and greater intraoperative blood loss were also associated with leakage. Protective stoma creation was associated with lower odds of leakage (adjusted OR, 0.614; 95% CI, 0.421–0.895; *p* = 0.011), whereas a low anastomotic level was associated with higher odds (adjusted OR, 1.701; 95% CI, 1.144–2.529; *p* = 0.009). Diabetes mellitus and neoadjuvant chemoradiotherapy were not significantly associated with leakage in this model (Table 4).

**Table 4:** Multivariable logistic regression analysis of factors associated with anastomotic leakage after propensity score matching.

Variable	*β*	SE	Adjusted OR	95% CI	*p* value
PNI, per 1-point increase	−0.108	0.021	0.898	0.861–0.936	<0.001
Diabetes mellitus, yes vs. no	0.221	0.196	1.247	0.849–1.832	0.260
Preoperative anemia, yes vs. no	0.563	0.188	1.756	1.214–2.540	0.003
Tumor distance from the anal verge, per 1-cm increase	−0.176	0.047	0.839	0.765–0.921	<0.001
Operative time, per 1-min increase	0.008	0.003	1.008	1.003–1.014	0.004
Intraoperative blood loss, per 1-mL increase	0.003	0.001	1.003	1.001–1.005	0.001
Protective stoma creation, yes vs. no	−0.488	0.193	0.614	0.421–0.895	0.011
Low anastomotic level, yes vs. no	0.531	0.203	1.701	1.144–2.529	0.009
Neoadjuvant chemoradiotherapy, yes vs. no	0.186	0.189	1.204	0.831–1.744	0.325

CI, confidence interval; OR, odds ratio; PNI, prognostic nutritional index; SE, standard error.

### 3.6 Analyses using alternative preoperative nutritional indicators

Analyses using alternative nutritional indicators showed associations in directions consistent with the primary PNI analysis. Each 1-g/L higher serum albumin concentration was associated with lower odds of anastomotic leakage (adjusted OR, 0.877; 95% CI, 0.828–0.930; *p* < 0.001), as was each 10-mg/L higher prealbumin concentration (adjusted OR, 0.931; 95% CI, 0.896–0.968; *p* < 0.001). Preoperative anemia was associated with higher odds of leakage (adjusted OR, 1.798; 95% CI, 1.246–2.595; *p* = 0.002) (Table 5).

**Table 5:** Analyses using alternative preoperative nutritional indicators after propensity score matching.

Model	Nutritional indicator	Adjusted OR	95% CI	*p* value
Model 1	PNI, per 1-point increase	0.898	0.861–0.936	<0.001
Model 2	Serum albumin, per 1-g/L increase	0.877	0.828–0.930	<0.001
Model 3	Prealbumin, per 10-mg/L increase	0.931	0.896–0.968	<0.001
Model 4	Preoperative anemia, yes vs. no	1.798	1.246–2.595	0.002

CI, confidence interval; OR, odds ratio; PNI, prognostic nutritional index.

### 3.7 Robustness analyses and discrimination of PNI

In the unmatched original cohort of 2,468 patients, including all 308 leakage events, the association between PNI and anastomotic leakage was similar to that observed after matching. Each 1-point increase in PNI was associated with an adjusted OR of 0.904 (95% CI, 0.882–0.927; *p* < 0.001), compared with an adjusted OR of 0.898 in the primary matched analysis. Associations for anemia, tumor distance from the anal verge, operative time, intraoperative blood loss, protective stoma creation, and low anastomotic level were also directionally consistent with those observed in the matched cohort (Supplementary Table S3).

The association between PNI and leakage remained statistically significant across sequentially adjusted models. The OR per 1-point increase in PNI was 0.866 (95% CI, 0.842–0.890) in the unadjusted model and 0.895 (95% CI, 0.859–0.933) after broader adjustment for demographic, clinical, tumor-related, and perioperative variables. After further adjustment for preoperative CRP, the association was attenuated but remained statistically significant (OR, 0.907; 95% CI, 0.870–0.945; *p* < 0.001). The corresponding E-value for the broadly adjusted estimate was 1.48, with an E-value of 1.35 for the confidence interval limit closest to the null; after additional CRP adjustment, these values were 1.44 and 1.31, respectively (Supplementary Table S4).

In the original cohort, the area under the receiver operating characteristic curve for preoperative PNI was 0.646 (95% CI, 0.611–0.681). A PNI cutoff of 45.5 maximized the Youden index (0.235), with a sensitivity of 62.7% and a specificity of 60.8% for distinguishing patients with and without postoperative anastomotic leakage (Supplementary Table S5 and Supplementary Figure S1).

### 3.8 Protective stoma and subgroup analyses

The association between protective stoma creation and anastomotic leakage was further examined according to anastomotic level in the original cohort. Among patients with an anastomotic level ≤5 cm, leakage occurred in 27.8% of patients without a protective stoma and 20.3% of those with a protective stoma; protective stoma creation was associated with lower adjusted odds of leakage in this subgroup (adjusted OR, 0.62; 95% CI, 0.44–0.87; *p* = 0.006). Among patients with an anastomotic level >5 cm, leakage occurred in 5.5 and 4.7% of patients without and with a protective stoma, respectively, and the adjusted association was not statistically significant (OR, 0.85; 95% CI, 0.45–1.60; *p* = 0.615). There was no statistically significant interaction between protective stoma creation and anastomotic level (P for interaction = 0.447) (Supplementary Table S6).

The inverse association between PNI and anastomotic leakage was also observed across the prespecified subgroups defined by neoadjuvant chemoradiotherapy, tumor location, protective stoma creation, diabetes mellitus, and tumor stage. Adjusted ORs per 1-point increase in PNI ranged from 0.886 to 0.911 across these strata, and none of the interaction tests was statistically significant (all P for interaction >0.05; Supplementary Table S7).

## 4 Discussion

This study provides a clinically focused evaluation of preoperative nutritional status in relation to postoperative anastomotic leakage after rectal cancer surgery. A notable strength of the present analysis is that PNI was not examined in isolation; its association was evaluated alongside NRS-2002, individual nutritional markers, CRP, PSM, unmatched-cohort regression, sequential adjustment, E-value analysis, and subgroup analyses. Across these complementary approaches, lower preoperative PNI was consistently associated with greater odds of anastomotic leakage. From a clinical perspective, PNI is attractive because it can be calculated from routinely available albumin and lymphocyte measurements without additional testing or specialized equipment (23, 24). The identified cutoff of 45.5 may therefore provide a pragmatic signal for preoperative risk stratification, although its modest discriminatory performance indicates that it should be interpreted as an adjunct rather than a stand-alone predictor. More broadly, the findings support incorporating nutritional and inflammatory assessment into multidimensional preoperative evaluation (25, 26). Patients with lower PNI or elevated nutritional risk may warrant closer review of nutritional reserve, inflammatory status, anemia, and other perioperative risk factors before surgery. Thus, the potential clinical value of PNI lies less in isolated prediction than in identifying patients who may benefit from more comprehensive preoperative assessment and individualized perioperative planning.

The nutritional findings showed a coherent pattern across several complementary indicators. Patients with anastomotic leakage had lower PNI, serum albumin, prealbumin, total protein, and lymphocyte counts, together with a higher prevalence of anemia and higher NRS-2002 scores. In adjusted analyses, PNI, albumin, and prealbumin were all inversely associated with leakage, whereas NRS-2002 showed a positive association when modeled either continuously or using the conventional cutoff of ≥3. This concordance suggests that the observed association reflects a broader impairment in nutritional and physiological reserve rather than an isolated abnormality in a single laboratory parameter. The quartile analysis further demonstrated a graded pattern, with leakage occurring in 19.0% of patients in the lowest PNI quartile compared with 7.1% in the highest quartile; the adjusted association was strongest in Q1, intermediate in Q2, and no longer statistically significant in Q3. Such a pattern is biologically plausible because successful anastomotic healing requires adequate substrate availability, collagen synthesis, tissue oxygenation, immune competence, and an appropriate response to surgical stress (27, 28). Nevertheless, PNI is not a purely nutritional construct. Serum albumin and lymphocyte count are both influenced by systemic inflammation, tumor-related metabolic changes, and immune status (29, 30). In this context, the higher CRP concentrations observed in patients with leakage are clinically relevant. Additional CRP adjustment attenuated the PNI estimate from 0.895 to 0.907, while the association remained statistically significant. This attenuation indicates that inflammatory activity may contribute to the relationship captured by PNI, supporting its interpretation as an integrated nutritional–inflammatory marker rather than a direct measure of malnutrition alone (31, 32).

The perioperative analyses also highlight the importance of anatomical and technical factors in the occurrence of anastomotic leakage. Greater tumor distance from the anal verge was associated with lower odds of leakage, whereas a low anastomotic level was associated with higher odds, consistent with the greater technical difficulty of low pelvic reconstruction and the potentially less favorable local perfusion and mechanical environment (33, 34). Longer operative time and greater intraoperative blood loss were also associated with leakage, findings that may reflect greater procedural complexity and physiological stress (35). Preoperative anemia was independently associated with higher odds of leakage, which is clinically plausible given the potential relevance of impaired oxygen delivery to tissue repair and anastomotic healing (36). Protective stoma creation was associated with lower odds of leakage in the primary model and remained associated with lower odds among patients with anastomoses ≤5 cm from the anal verge. However, the formal interaction between protective stoma creation and anastomotic level was not statistically significant. Therefore, these findings do not support a conclusion that the association differs materially according to anastomotic height, and the stoma result should remain interpreted as an observational association. The PNI association was also directionally consistent across subgroups defined by neoadjuvant chemoradiotherapy, tumor location, protective stoma creation, diabetes mellitus, and tumor stage, with no significant interactions. In addition, the similar effect estimates obtained in the matched and unmatched cohorts suggest that the observed association was not driven solely by the matching procedure and was relatively stable across different analytical populations.

Wei et al. (37) conducted a retrospective study of patients undergoing colorectal cancer surgery and reported that lower preoperative PNI was associated with anastomotic leakage; however, the discriminatory ability of PNI alone was modest, with an AUC of 0.625. This finding is closely aligned with our results, in which PNI showed an AUC of 0.646, suggesting that although PNI is associated with leakage, its value as an isolated predictive marker remains limited. Shayimu et al. (38) evaluated 560 patients undergoing laparoscopic rectal cancer surgery and found that preoperative albumin and prealbumin, together with tumor location and intraoperative bleeding, were associated with anastomotic leakage. These observations are consistent with our findings that lower albumin and prealbumin levels, shorter tumor distance from the anal verge, and greater intraoperative blood loss were associated with higher odds of leakage, highlighting the associations of both nutritional and operative characteristics with anastomotic leakage. More recently, Kor et al. (39) retrospectively analyzed 2,026 patients undergoing curative-intent colorectal cancer surgery using PSM and reported that higher PNI was associated with substantially lower odds of anastomotic leakage (adjusted OR, 0.16), further supporting an association between preoperative immunonutritional status and postoperative anastomotic leakage. Unlike the dichotomized PNI approach used by Kor et al., our primary analysis modeled PNI continuously and demonstrated similar estimates in the matched cohort (OR, 0.898 per 1-point increase) and the complete unmatched cohort (OR, 0.904), providing complementary evidence that the observed association was relatively consistent across analytical populations. Overall, these studies and our findings collectively support PNI as a readily available component of preoperative risk assessment, while its modest standalone discrimination indicates that it is more appropriately interpreted alongside other nutritional, inflammatory, anatomical, and perioperative factors.

Several limitations should be considered. First, the retrospective, single-center design may have introduced selection bias and residual confounding despite PSM and multivariable adjustment. Matching reduced the number of non-leakage observations and may have limited precision and representativeness, although all 308 leakage events were retained and the similar PNI estimates in the matched and unmatched cohorts support the consistency of the main association. Second, unmeasured confounding cannot be excluded. The E-value analysis quantified the strength of confounding required to explain the observed association but cannot eliminate this possibility. Third, PNI is an indirect marker reflecting both nutritional and inflammatory status. Body-composition measures and related clinical information, including skeletal muscle mass, validated sarcopenia assessment, recent weight loss, dietary intake, and frailty, were not routinely documented and could not be reliably analyzed. Accordingly, the observed association should not be attributed solely to nutritional status, and PNI should not be considered a surrogate for sarcopenia. Fourth, the observational design precludes causal inference regarding whether improving PNI would reduce anastomotic leakage. The data-derived PNI cutoff of 45.5 also showed only modest discrimination and should therefore be considered exploratory. Finally, this study focused on 30-day leakage and did not evaluate long-term oncologic or functional outcomes. Future prospective multicenter studies with external validation should incorporate standardized nutritional assessment, inflammatory markers, body-composition and frailty measures, and longer-term follow-up, and should assess whether combining PNI with anatomical, operative, and clinical factors improves perioperative risk stratification.

## 5 Conclusion

Lower preoperative PNI was independently associated with higher odds of postoperative anastomotic leakage in patients undergoing rectal cancer surgery. Lower albumin and prealbumin levels, preoperative anemia, and higher NRS-2002 scores showed concordant associations, while the association between PNI and leakage remained consistent across matched and unmatched analyses, sequential adjustment, additional CRP adjustment, and clinical subgroups. Shorter tumor distance from the anal verge, longer operative time, greater intraoperative blood loss, and low anastomotic level were also associated with higher odds of leakage. These findings support the potential value of PNI as an adjunct to multidimensional preoperative risk assessment, rather than as a stand-alone predictor, in patients undergoing rectal cancer surgery.

## Data Availability

The raw data supporting the conclusions of this article will be made available by the authors, without undue reservation.
