# Supplementary material for: Association between preoperative nutritional status and anastomotic leakage in patients undergoing rectal cancer surgery
Source: Front Nutr. 2026 Oct 28;13:1932994. doi: 10.3389/fnut.2026.1932994 (PMC13752076; doi:10.3389/fnut.2026.1932994)
Supplement: Supplementary file 1 [file Supplementary_file_1.DOCX]

Supplementary Table S1. Comparison of the source non-leakage cohort and propensity score-matched controls

| Variable | Source non-leakage cohort (n = 2160) | Matched controls (n = 308) | SMD |
| --- | --- | --- | --- |
| Age, years | 63.4 ± 9.7 | 66.5 ± 8.9 | 0.333 |
| Male sex, n (%) | 1364 (63.1) | 205 (66.6) | 0.071 |
| BMI, kg/m² | 24.2 ± 3.3 | 23.5 ± 3.1 | 0.219 |
| Tumor location, n (%) |  |  | 0.176 |
| └ Low rectum | 864 (40.0) | 150 (48.7) |  |
| └ Mid rectum | 842 (39.0) | 112 (36.4) |  |
| └ High rectum | 454 (21.0) | 46 (14.9) |  |
| Neoadjuvant chemoradiotherapy, n (%) | 629 (29.1) | 114 (37.0) | 0.168 |
| Surgical approach, n (%) |  |  | 0.183 |
| └ Open surgery | 410 (19.0) | 82 (26.6) |  |
| └ Laparoscopic surgery | 1512 (70.0) | 203 (65.9) |  |
| └ Robotic surgery | 238 (11.0) | 23 (7.5) |  |
| ASA III–IV, n (%) | 734 (34.0) | 142 (46.1) | 0.249 |
| Diabetes mellitus, n (%) | 410 (19.0) | 81 (26.3) | 0.176 |
| Protective stoma creation, n (%) | 756 (35.0) | 138 (44.8) | 0.201 |
| Tumor stage, n (%) |  |  | 0.257 |
| └ Stage I | 540 (25.0) | 55 (17.9) |  |
| └ Stage II | 907 (42.0) | 113 (36.7) |  |
| └ Stage III | 713 (33.0) | 140 (45.5) |  |

Abbreviations: ASA, American Society of Anesthesiologists; BMI, body mass index; SMD, standardized mean difference.

Supplementary Table S2. Association between Nutritional Risk Screening 2002 and anastomotic leakage after propensity score matching

| NRS-2002 variable | Leakage group (n = 308) | Non-leakage group (n = 308) | Adjusted OR | 95% CI | P value |
| --- | --- | --- | --- | --- | --- |
| NRS-2002 score, median (IQR) | 2 (1–3) | 1 (1–2) | 1.31 per 1-point increase | 1.12–1.54 | <0.001 |
| NRS-2002 <3, n (%) | 226 (73.4) | 260 (84.4) | 1.00 (reference) | — | — |
| NRS-2002 ≥3, n (%) | 82 (26.6) | 48 (15.6) | 1.72 | 1.12–2.65 | 0.013 |

Abbreviations: CI, confidence interval; IQR, interquartile range; NRS-2002, Nutritional Risk Screening 2002; OR, odds ratio.

Supplementary Table S3. Multivariable analysis of factors associated with anastomotic leakage in the unmatched cohort (n = 2468; 308 leakage events)

| Variable | Adjusted OR | 95% CI | P value |
| --- | --- | --- | --- |
| PNI, per 1-point increase | 0.904 | 0.882–0.927 | <0.001 |
| Diabetes mellitus, yes vs. no | 1.214 | 0.913–1.614 | 0.182 |
| Preoperative anemia, yes vs. no | 1.642 | 1.264–2.133 | <0.001 |
| Tumor distance from the anal verge, per 1-cm increase | 0.866 | 0.823–0.912 | <0.001 |
| Operative time, per 1-min increase | 1.006 | 1.003–1.009 | <0.001 |
| Intraoperative blood loss, per 1-mL increase | 1.002 | 1.001–1.003 | <0.001 |
| Protective stoma creation, yes vs. no | 0.658 | 0.505–0.858 | 0.002 |
| Low anastomotic level, yes vs. no | 1.612 | 1.231–2.112 | <0.001 |
| Neoadjuvant chemoradiotherapy, yes vs. no | 1.163 | 0.894–1.513 | 0.260 |

Abbreviations: CI, confidence interval; OR, odds ratio; PNI, prognostic nutritional index.

Supplementary Table S4. Sequentially adjusted and inflammation-adjusted analyses of the association between PNI and anastomotic leakage after propensity score matching (n = 616)

| Model | Adjustment variables | OR for PNI per 1-point increase | 95% CI | P value | E-value for estimate | E-value for CI limit |
| --- | --- | --- | --- | --- | --- | --- |
| Model 1 | Unadjusted | 0.866 | 0.842–0.890 | <0.001 | 1.58 | 1.50 |
| Model 2 | Age, sex, and BMI | 0.875 | 0.850–0.901 | <0.001 | 1.55 | 1.46 |
| Model 3 | Model 2 + diabetes mellitus, preoperative anemia, tumor distance from the anal verge, operative time, intraoperative blood loss, protective stoma creation, low anastomotic level, and neoadjuvant chemoradiotherapy | 0.895 | 0.859–0.933 | <0.001 | 1.48 | 1.35 |
| Model 4 | Model 3 + preoperative CRP | 0.907 | 0.870–0.945 | <0.001 | 1.44 | 1.31 |

Abbreviations: BMI, body mass index; CI, confidence interval; CRP, C-reactive protein; OR, odds ratio; PNI, prognostic nutritional index.

Supplementary Table S5. Receiver operating characteristic analysis of preoperative PNI for anastomotic leakage in the original cohort

| Performance measure | Estimate |
| --- | --- |
| Analysis population | Original cohort, n = 2468 |
| AUC | 0.646 |
| 95% CI | 0.611–0.681 |
| Optimal PNI cutoff | 45.5 |
| Sensitivity | 62.7% |
| Specificity | 60.8% |
| Youden index | 0.235 |

Abbreviations: AUC, area under the receiver operating characteristic curve; CI, confidence interval; PNI, prognostic nutritional index.

Supplementary Table S6. Association between protective stoma creation and anastomotic leakage according to anastomotic level in the original cohort

| Anastomotic level | Protective stoma | Total, n | AL, n (%) | Adjusted OR for protective stoma | 95% CI | P value | P for interaction |
| --- | --- | --- | --- | --- | --- | --- | --- |
| ≤5 cm | No | 360 | 100 (27.8) | 1.00 (reference) | — | — |  |
| ≤5 cm | Yes | 640 | 130 (20.3) | 0.62 | 0.44–0.87 | 0.006 | 0.447 |
| >5 cm | No | 1210 | 66 (5.5) | 1.00 (reference) | — | — |  |
| >5 cm | Yes | 258 | 12 (4.7) | 0.85 | 0.45–1.60 | 0.615 |  |

Abbreviations: AL, anastomotic leakage; CI, confidence interval; OR, odds ratio.

Supplementary Table S7. Subgroup analyses of the association between PNI and anastomotic leakage after propensity score matching

| Subgroup | n | Adjusted OR per 1-point increase in PNI | 95% CI | P value | P for interaction |
| --- | --- | --- | --- | --- | --- |
| Neoadjuvant chemoradiotherapy |  |  |  |  | 0.418 |
| Yes | 232 | 0.886 | 0.832–0.943 | <0.001 |  |
| No | 384 | 0.905 | 0.860–0.952 | <0.001 |  |
| Tumor location |  |  |  |  | 0.287 |
| Low rectum | 306 | 0.892 | 0.845–0.942 | <0.001 |  |
| Mid/high rectum | 310 | 0.911 | 0.858–0.967 | 0.002 |  |
| Protective stoma creation |  |  |  |  | 0.534 |
| Yes | 280 | 0.903 | 0.849–0.961 | 0.001 |  |
| No | 336 | 0.894 | 0.847–0.944 | <0.001 |  |
| Diabetes mellitus |  |  |  |  | 0.621 |
| Yes | 166 | 0.887 | 0.812–0.970 | 0.008 |  |
| No | 450 | 0.902 | 0.861–0.945 | <0.001 |  |
| Tumor stage |  |  |  |  | 0.473 |
| Stage I–II | 336 | 0.907 | 0.857–0.959 | 0.001 |  |
| Stage III | 280 | 0.891 | 0.842–0.943 | <0.001 |  |

Abbreviations: CI, confidence interval; OR, odds ratio; PNI, prognostic nutritional index.

Supplementary Figure S1. Receiver operating characteristic curve of preoperative PNI for anastomotic leakage
